# Skin hypersensitivity in chronic cough patients: symptom profiles and psychosomatic correlates

**DOI:** 10.1080/07853890.2026.2638047

**Published:** 2026-03-09

**Authors:** Tongyangzi Zhang, Heng Wu, Haodong Bai, Jiguang Wu, Lili Zhang, Rongrong Li, Yiqing Zhu, Bingxian Sha, Jiaying Yuan, Yaxing Zhou, Xianghuai Xu, Li Yu

**Affiliations:** a Department of Pulmonary and Critical Care Medicine, Tongji Hospital, School of Medicine, Tongji University, Shanghai, China; b Department of Psychosomatic Medicine, Tongji Hospital, School of Medicine, Tongji University, Shanghai, China; c Department of Dermatology, Tongji Hospital, School of Medicine, Tongji University, Shanghai, China

**Keywords:** Chronic cough, sensitive skin syndrome, psychosomatic co-morbidity, refractory chronic cough, neuromodulator

## Abstract

**Background:**

Cough hypersensitivity syndrome (CHS) is a characteristic of patients with chronic cough (CC). Sensitive skin syndrome (SSS), which is characterised by cutaneous pain and pruritus, may share neural hypersensitivity mechanisms with CHS. This study aimed to determine the co-morbidities, clinical profiles, and psychosomatic correlates in CC patients.

**Methods:**

Two hundred CC patients were enrolled in this prospective cohort study. SSS was diagnosed according to established guidelines, which required the presence of subjective symptoms induced by minimal stimuli with at least one of the following positive criteria: Sensitive Scale-10 score > 13; Sensitive Scale-14 score > 18; lactic acid sting test score ≥ 3; or capsaicin test score ≥ 3. Assessments included cough severity, Visual Analogue Scale (VAS), capsaicin cough sensitivity, cough symptom score, Leicester cough questionnaire (LCQ), and psychological evaluations.

**Results:**

Among CC patients, 44.5% (89/200) had SSS with a higher prevalence in refractory/unexplained CC (RU-CC) patients compared to non-RU-CC patients (63.24% vs. 34.85%; *p* < 0.001). SSS patients exhibited heightened cough sensitivity (lower C2/C5 thresholds; *p* = 0.017/0.004), higher VAS scores (*p* = 0.026), lower LCQ scores, and an elevated psychological burden compared to non-SSS patients. In addition, RU-CC patients with SSS had superior cough responses to neuromodulators than non-SSS patients (LCQ improvement: 2.59 ± 2.36 vs. 1.26 ± 2.53; *p* = 0.037; response rate: 79.3% vs. 44.4%; *p* = 0.029).

**Conclusion:**

SSS was identified in a clinically relevant subset of CC patients (especially those with RU-CC) and correlated with neural hypersensitivity and psychological distress. Early recognition of SSS in patients with CC and the early introduction of neuromodulators may offer greater therapeutic benefits and improve patient outcomes.

## Introduction

Cough is one of the most common complaints in respiratory outpatient clinics. Chronic cough (CC), defined as a persistent cough lasting > 8 weeks without radiologic abnormalities, affects approximately 9.6% of the general population [1]. Of these cases, a significant proportion are classified as refractory or unexplained chronic cough (RU-CC), a condition that persists despite thorough evaluation and treatment [2,3], imposing a substantial physical and psychological burden on patients and representing a major challenge in clinical practice [4,5]. The cough hypersensitivity syndrome (CHS) has been widely adopted to explain the underlying pathophysiology, conceptualising CC as a state of heightened cough reflex sensitivity driven by both peripheral and central neural sensitisation [6].

Interestingly, a parallel hypersensitivity phenomenon exists in dermatology as sensitive skin syndrome (SSS), a common condition reported by over 50% of the population in certain regions, which is characterised by unpleasant sensations such as stinging, burning, and itching in response to normally innocuous stimuli [7–9]. Similar to CHS, the pathophysiology of SSS is believed to involve dysregulation of cutaneous sensory nerves, including impairment of peripheral C-fibres and amplification of sensory signals within the central nervous system [10–14].

Notably, converging evidence indicates that pain, itch, and cough, all being protective sensory reflexes, may share overlapping neurobiological mechanisms [15–17], suggesting a potential link between SSS and CHS. Furthermore, SSS has also been strongly linked to a high prevalence of psychological disorders, such as anxiety and depression [9]. However, beyond anxiety and depression, somatic symptom disorder (SSD), which is characterised by persistent and distressing somatic complaints accompanied by excessive thoughts, feelings, or behaviours related to these symptoms, may be particularly relevant in patients with multisensory hypersensitivity. Evaluating the role of this disorder in CC patients with and without SSS may therefore help clarify the link between these two hypersensitivity conditions.

However, while prior epidemiologic studies have indicated an association between chronic cough and skin sensitivity symptoms [18–21], the studies were primarily based on general population databases using non-specific definitions and lacked objective, detailed cough assessments. To date, no study has prospectively and systematically evaluated SSS as a comorbidity in a well-characterised cohort of CC patients using established dermatologic, respiratory, and psychosomatic assessments.

Therefore, this study aimed to determine the prevalence of SSS in CC patients and to investigate whether those with SSS represent a unique subgroup with distinct clinical profiles and treatment responses. We compared them to CC patients without SSS in terms of cough reflex sensitivity, quality of life, and psychosomatic correlates to elucidate the mechanisms underlying CHS and explore potential therapeutic strategies.

## Material and methods

### Study design

This was an observational study which was registered in the Chinese Clinical Trials Register (http://www.chictr.org.cn/ [ChiCTR2400083115]). The study was conducted in accordance with the Declaration of Helsinki and was approved by the Ethics Committee of Tongji Hospital (Approval Number: K-2024-005). Written informed consent was obtained from all participants prior to enrollment. Patients with CC underwent a comprehensive etiologic evaluation based on clinical history and a series of diagnostic investigations, including chest computed tomography, complete blood count, serum total immunoglobulin E (IgE), routine pulmonary function tests, fractional exhaled nitric oxide (FeNO), and induced sputum cytology. Additional assessments, including bronchial provocation testing, multichannel intraluminal impedance-pH monitoring, and bronchoscopy, were performed in select cases. Common comorbidities associated with chronic cough were also recorded based on patient history, previous diagnoses, and available medical records.

During the initial consultation, all patients underwent a comprehensive and standardised assessment protocol in a dedicated research setting. This assessment encompassed three main components: (1) evaluation of cough sensitivity and cough-related quality of life; (2) assessment of skin sensitivity; and (3) a psychological evaluation. General demographic data, examination results, and questionnaire-based assessment data were collected. Patients received etiologic or empirical treatment with follow-up assessments performed biweekly to determine the underlying cause and evaluate treatment outcomes.

### Participants

The inclusion criteria were as follows: (1) age ≥ 18 years; (2) diagnosis of CC in accordance with the *Chinese National Guideline on Diagnosis and Management of Cough (2021)* [22], and (3) completion of both cough-related evaluations and psychosomatic assessments during the initial visit.

The exclusion criteria were as follows: (1) current smoking or abstinence for < 2 years; (2) inability to complete questionnaires due to language or cognitive barriers; and (3) non-completion of required baseline evaluations.

SSS was defined according to the *Chinese Clinical Guidelines for the Diagnosis and Treatment of Sensitive Skin* (2024 edition) [23]. Patients were required to fulfil the primary condition to satisfy the following diagnostic criteria: the presence of subjective symptoms, such as burning, transient erythema (flushing), stinging, itching, or a feeling of tightness in response to minimal external stimuli with or without persistent facial erythema. In addition, at least one of the following secondary criteria had to be met: (1) abnormal scores on standardized sensitivity questionnaires (Sensitive Scale-10 [SS-10] score > 13 or Sensitive Scale-14 [SS-14] score > 18); (2) a score ≥ 3 on the lactic acid sting test (LAST) or the capsaicin test (CAT); and (3) presence of facial dermatoses, such as rosacea, seborrheic dermatitis, steroid-dependent dermatitis, contact dermatitis, atopic dermatitis, or tumid lupus erythematosus, accompanied by clinical symptoms of sensitive skin, in which case the diagnosis was considered secondary sensitive skin. Patients combined with SSS were defined as the SSS group, while patients without SSS were defined as the non-SSS (NSSS) group. The severity of SSS was assessed based on the SS-14 score. Patients who were only LAST- and/or CAT-positive were defined as the tolerable group.

The diagnosis and management of CC, including the identification of refractory or unexplained chronic cough (RU-CC), were performed by respiratory physicians in accordance with the Chinese guidelines for the diagnosis and treatment of cough [22].

Based on the clarity of the etiology and treatment response, patients were defined as RU-CC and classified into three categories: (1) RCC, which was defined by positive findings on auxiliary tests and satisfactory response only when neuromodulators are combined with etiologic treatment, (2) unexplained CC (UCC) in which the patient responded poorly even with the addition of neuromodulators despite positive findings, and (3) UCC, in which no clear etiology was identified after a comprehensive evaluation [24].

The diagnosis of psychological disorders, including anxiety, depression, and somatic symptom disorder (SSD), was established based on clinical evaluation and standardized psychological scales with confirmation provided by the psychosomatic medicine team through a multidisciplinary consultation.

### Cough sensitivity assessment

Cough severity was assessed using the cough severity visual analogue scale (VAS), which has a 0–100 mm scale with higher scores indicating a more severe cough [25]. Cough symptom scores were evaluated using the Hsu scale, which includes separate day- and night-time components, each ranging from 0 (no cough) to 5 (severe cough) [26]. Cough-related quality of life was measured using the validated Chinese version of the Leicester cough questionnaire (LCQ), a 19-item instrument covering physical, psychological, and social domains [27]. Cough reflex sensitivity was assessed *via* the capsaicin cough challenge test in accordance with European Respiratory Society guidelines, with C2 and C5 defined as the lowest capsaicin concentrations that induced ≥ 2 and ≥ 5 coughs, respectively [28].

### Skin sensitivity symptom assessment

Skin sensitivity was assessed using a combination of validated scales and provocation tests. SS-10: The SS-10 evaluates eight common subjective symptoms of sensitive skin, including stinging, burning, itching, tightness, and redness. Each symptom is rated from 0 (none) to 10 (unbearable), with a total score > 13 indicating sensitive skin and > 5 suggesting mild sensitivity [29]. SS-14: The SS-14 is a 14-item questionnaire that was adapted from the Baumann Sensitive Skin Questionnaire and is tailored to the Chinese population. Eleven items assess sensitivity triggers (scored from 1–4) and three items address personal and family history of allergies or facial dermatoses (scored 1 or 2). Total scores are categorised as tolerant (12–17), mild (18–23), moderate (24–32), and severe sensitivity (33–42) [23]. LAST: At room temperature, 50 μL of a 10% lactic acid solution was applied to the nasolabial fold and to one side of the cheek. The subject was asked about subjective symptoms at 2.5 and 5 min and scored using a 4-point scale (0 = none, 3 = severe); a total score ≥ 3 was defined as a positive result [30]. CAT: Two layers of filter paper (diameter, 0.8 cm) were placed approximately 1 cm lateral to one side of the nasolabial fold and on one side of the cheek. Then, 50 μL of 0.001% capsaicin solution was applied to the filter paper. Subjects were asked to report sensory perceptions and rate the intensity of the burning sensation on a 5-point scale [1 = barely perceptible, 5 = painful]. A score ≥ 3 lasting > 30 s was considered positive [23].

### Psychological related assessment

Psychological status was assessed using a set of standardised self-report questionnaires: Generalised Anxiety Disorder-7 (GAD-7): GAD-7 is a 7-item scale used to screen for anxiety severity. The total score is 21 points and a score ≥ 5 suggests abnormalities [31]. Patient Health Questionnaire-9 (PHQ-9): PHQ-9 is a 9-item instrument evaluating depressive symptoms. The total score ranges from 0–27; a score > 5 suggests abnormalities [32]. Somatic Symptom Disorder-12 (SSD-12): The SSD-12 is used to assess cognitive and emotional aspects of somatic symptom disorders. The total score is 48 points; a higher score indicates a more severe reaction to somatic symptoms. A score ≥ 16 is divided into the threshold value and the psychometric evaluation has indicated good reliability and validity [33]. Patient Health Questionnaire-15 (PHQ-15): The PHQ-15 measures the severity of somatic symptoms and is comprised of 15 somatic symptoms, each scored from 0 (“not bothered at all”) to 2 (“bothered a lot”), with a total score of 30 points. It has been shown that the Chinese version of the PHQ‐15 exhibits satisfactory reliability and provides preliminary evidence of validity [34].

### Data analysis

Normally distributed data are expressed as the mean ± standard deviation, whereas data with skewed distributions are expressed as a median (interquartile range). C2 and C5 were log-transformed to normalise the data. Categorical variables were analysed with the chi‐square test. Continuous variables that were normally distributed were evaluated with the *t*‐test. Otherwise, the Mann‐Whitney nonparametric test was used. SPSS 26.0 software (SPSS Inc., Chicago, IL, USA) was used for all statistical analyses. A *P*-value < 0.05 was accepted as statistically significant.

## Results

### Participants characteristics

**Figure 1.**
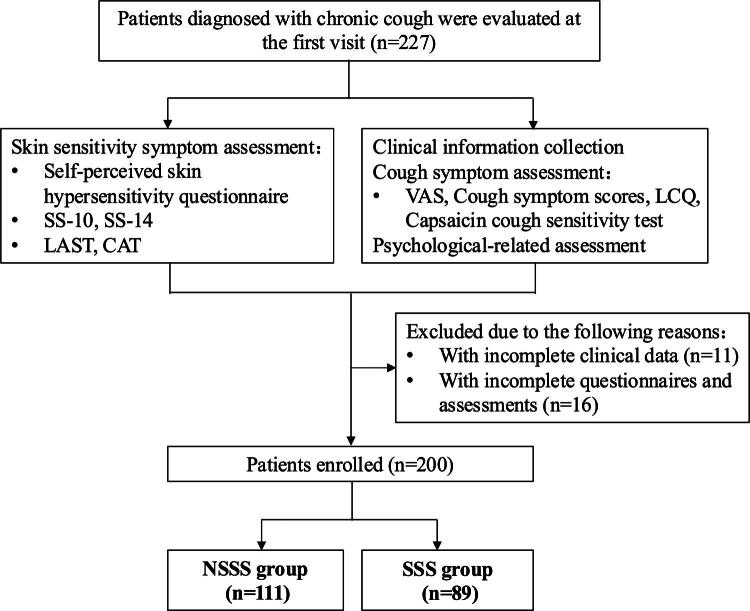
Patient flow through the study.

Between May 2024 and March 2025, a total of 227 patients with chronic cough were screened for eligibility. After exclusion of 27 patients who did not complete the required baseline questionnaires and assessments, 200 patients were included in the final analysis (Figure 1). There was no significant difference between the SSS and NSSS groups with respect to sex, age, disease duration, body mass index (BMI), peripheral blood eosinophil count, eosinophils in induced sputum, serum IgE level, FeNO, or pulmonary function parameters (Table 1).

**Table 1.** General clinical characteristics of enrolled patients with chronic cough.

Variables	NSSS (*N* = 111)	SSS (*N* = 89)	Test results
Age (y)	50.69 ± 15.93	49.06 ± 15.74	*t* = −0.726, *p* = 0.469
Sex (F/M)	70/41	58/31	χ^2^ = 0.095, *p* = 0.758
BMI (kg/m^2^)	24.19 ± 3.95	24.04 ± 4.03	*t* = 0.260, *p* = 0.795
Duration (m)	12.00 (44.00)	12.00 (44.50)	Z = −0.105, *p* = 0.917
Lung function			
FEV1 predicted (%)	99.08 ± 15.91	98.67 ± 19.66	*t* = 0.096, *p* = 0.924
FVC predicted (%)	99.61 ± 15.65	100.38 ± 17.17	*t* = −0.198, *p* = 0.844
FEV1/FVC%	80.45 ± 9.83	80.41 ± 8.08	*t* = 0.021, *p* = 0.983
FeNO (ppb)	23.16 ± 22.81	23.73 ± 30.53	*t* = −0.151, *p* = 0.880
IgE (IU/mL)	74.52 ± 128.52	92.58 ± 164.45	*t* = −0.848, *p* = 0.397
PBEC (×10^9^/L)	1.15 ± 1.69	1.24 ± 2.00	*t* = −0.351, *p* = 0.726
sEOS%	0.00 (2.00)	1.00 (2.00)	Z = −0.912, *p* = 0.362

Data are presented as the mean ± standard deviation, median (interquartile range), or No. (%) unless otherwise indicated.

NSSS, non-sensitive skin syndrome; SSS, sensitive skin syndrome; BMI, body mass index; FEV1, forced expiratory volume in 1 s; FVC, forced vital capacity; FeNO, fractional exhaled nitric oxide; IgE, immunoglobulin E; PBEC, peripheral blood eosinophil counts; sEOS%, eosinophils in induced sputum.

### Prevalence of SSS in CC

**Figure 2.**
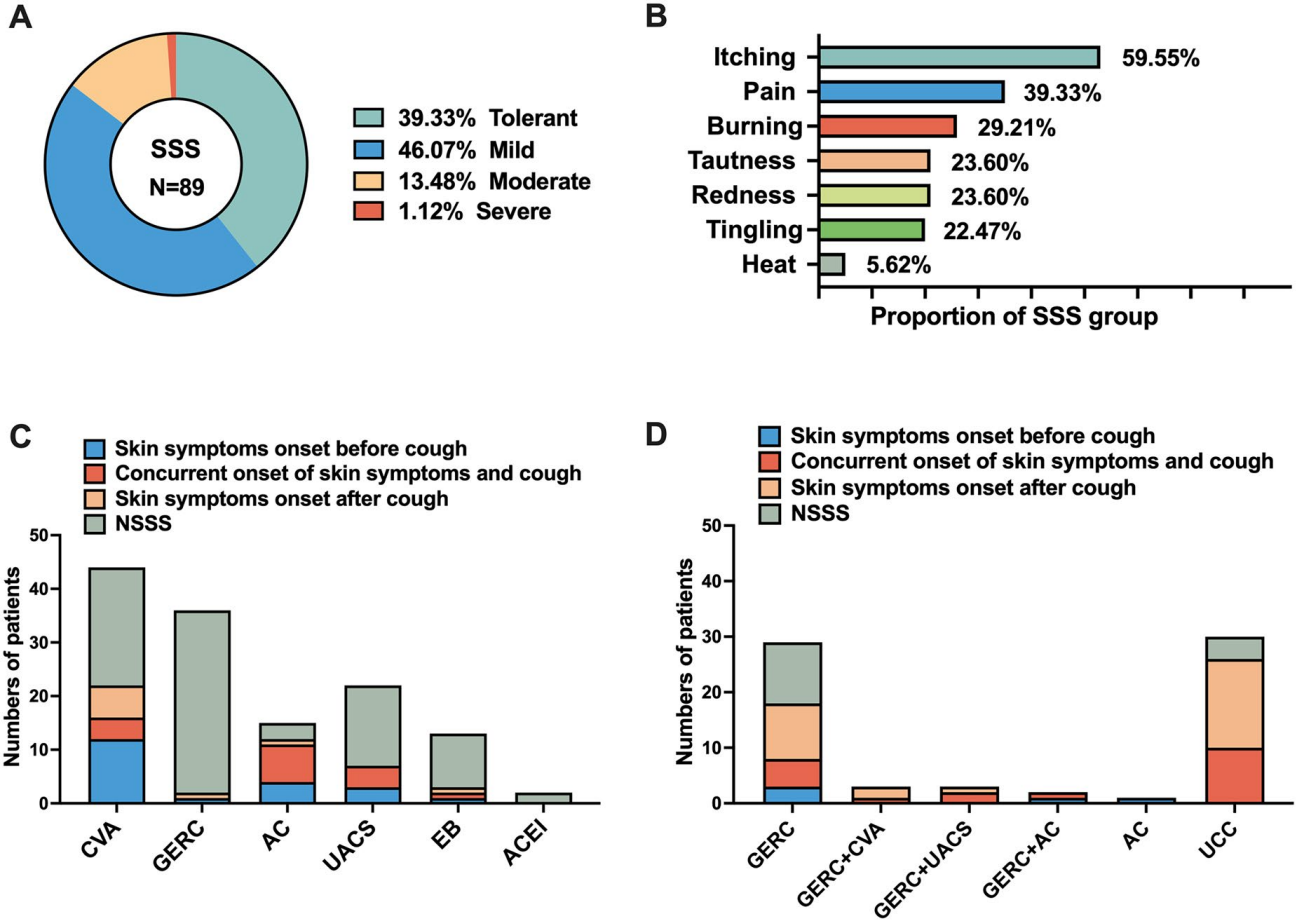
Characteristics of skin symptoms in chronic cough patients. (A) Severity of skin symptoms in the SSS group; (B) characteristics of skin symptoms in the SSS group; (C) aetiology and symptom onset in non-RU-CC with SSS; and (D) aetiology and symptom onset in RU-CC with SSS.

Among the 200 patients with CC, 89 (44.5%) exhibited skin hypersensitivity, including 54 with abnormal scores on standardised sensitivity questionnaires and 35 who tested positive on the LAST and/or CAT assessments. Based on SS-14 scoring, 35 patients were classified as tolerant, 41 had mild sensitivity, 12 had moderate sensitivity, and one had severe sensitivity (Figure 2A). The most frequently reported skin symptoms in the SSS group were itching (59.55%, 53/89) and pain (39.33%, 35/89), followed by burning (29.21%), tautness (23.60%), redness (23.60%), tingling (22.47%), and heat (5.62%) (Figure 2B).

The prevalence of SSS varied significantly between clinical subgroups. It was observed in 34.85% (46/132) of patients with non-RU-CC, compared to 63.24% (43/68) of those with RU-CC. Among non-RU-CC patients with SSS, the most common etiologies were cough variant asthma (CVA) (22/46), atopic cough (AC) (12/46), and upper airway cough syndrome (UACS) (7/46). In this subgroup, skin symptoms were more likely to precede or coincide with the onset of cough (Figure 2C). While, among RU-CC patients with SSS, the predominant etiologies were refractory gastroesophageal reflux-related cough (GERC) (18/43) and unexplained chronic cough (UCC) (26/43). Notably, in this group, the onset of cough frequently preceded the development of skin symptoms (Figure 2D).

The prevalence of key comorbidities in the overall cohort and by SSS status is detailed in Supplementary Table 1. In brief, aside from a significantly higher prevalence of nasal disorders and allergic skin diseases in the SSS group compared to the NSSS group (both *p* < 0.05), there were no significant differences in the frequency of other conditions, including hypertension, diabetes, cardiac diseases, or thyroid disorders. Notably, within the SSS group, 29 patients (10 males and 19 females) self-reported specific allergic skin conditions, including 14 with eczema, five with allergic dermatitis, and 10 with urticaria or related disorders.

### Clinical and psychological profile of CC patients with and without SSS

Patients in the SSS group exhibited significantly lower C2 and C5 thresholds compared to the NSSS group (C2: *p* = 0.017; C5: *p* = 0.004), indicating heightened capsaicin cough sensitivity. Additionally, the SSS group reported higher VAS scores (*p* = 0.026) and lower LCQ total (*p* = 0.004) and all domain scores (Physical: *p* = 0.040; Psychological: *p* < 0.001; Social: *p* = 0.002). However, there was no significant difference in cough symptom scores (Daytime: *p* = 0.951; Nighttime: *p* = 0.705). Notably, the proportion of patients with RU-CC was significantly higher in the SSS group compared to the NSSS group (*p* < 0.001; Table 2).

**Table 2.** Comparison of cough and psychological evaluations between the NSSS and SSS patients with chronic cough.

	Variables	NSSS (*N* = 111)	SSS (*N* = 89)	Test results
Cough-related assessment	RU-CC (Yes/No)	25/86	43/46**	χ^2^ = 14.643, *p* < 0.001
	VAS	59.65 ± 20.70	64.83 ± 16.82*	*t* = −1.954, *p* = 0.026
	Cough symptom score			
	Daytime	3.00 (1.00)	3.00 (1.00)	Z = −0.061, *p* = 0.951
	Nighttime	2.00 (1.00)	2.00 (1.00)	Z = −0.378 *p* = 0.705
	Capsaicin cough sensitivity			
	lgC2	0.91 ± 0.39	0.81 ± 0.19*	*t* = 2.417, *p* = 0.017
	lgC5	1.06 ± 0.61	1.89 ± 0.30*	*t* = 2.895, *p* = 0.004
	LCQ score			
	Total score	14.96 (5.23)	12. 93 (5.42)*	Z = −2.911, *p* = 0.004
	Physical domain	4.88 (1.63)	4.50 (1.88)*	Z = −2.264, *p* = 0.040
	Psychological domain	5.14 (2.14)	3.86 (1.50)**	Z = −4.437, *p* < 0.001
	Social domain	5.14 (2.14)	4.29 (2.14)*	Z = −3.171, *p* = 0.002
Psychological assessment	SSD-12 score	4.0 (12.0)	16.0 (11.5)**	Z = −5.154, *p* < 0.001
	PHQ-15 score	2.0 (5.0)	5.0 (6.0)**	Z = −4.417, *p* < 0.001
	GAD-7 score	1.0 (4.0)	4.0 (7.0)**	Z = −3.767, *p* < 0.001
	PHQ-9 score	2.0 (5.0)	4.0 (5.0)**	Z = −3.071, *p* = 0.002

Data are presented as the mean ± SD, median (interquartile range), or No. (%) unless otherwise indicated.

RU-CC, refractory chronic cough or unexplained chronic cough; NSSS, non-sensitive skin syndrome; SSS, sensitive skin syndrome; VAS, cough severity visual analog scale; C2, capsaicin solution concentration with ≥ 2 coughs; C5, capsaicin solution concentration with ≥ 5 coughs; SSD-12, somatic symptom disorder-B criteria scale; LCQ, Leicester cough questionnaire; PHQ-15, Patient Health Questionnaire-15; PHQ-9, Patient Health Questionnaire-9; GAD-7, General Anxiety Disorder-7.

*Compared to the NSSS group, *p* < 0.05; **compared to the NSSS group, *p* < 0.001.

**Figure 3.**
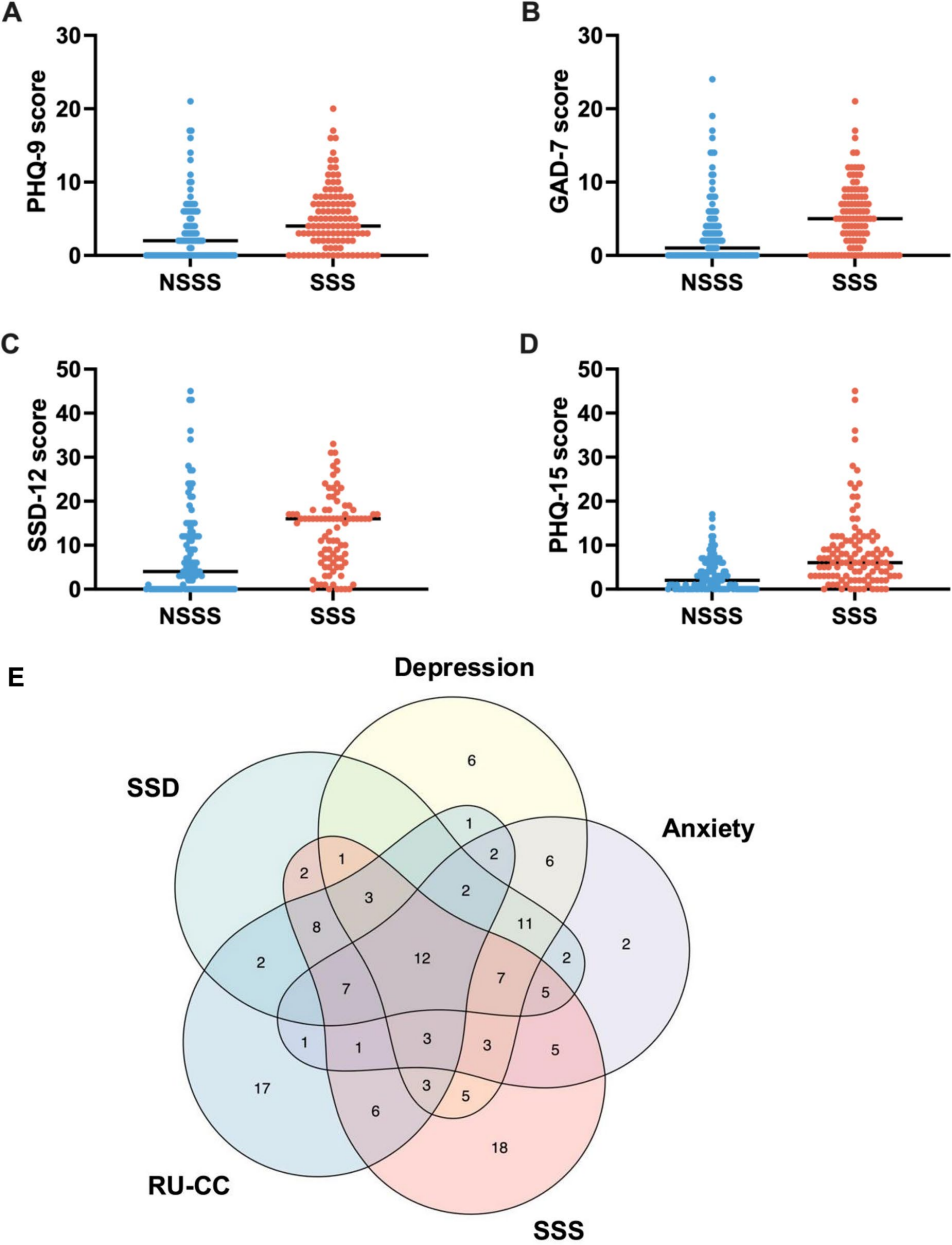
Comparison of psychological assessments between the SSS and NSSS groups. (A) Comparison of PHQ-9 scores between the SSS and NSSS groups; (B) comparison of GAD-7 scores between the SSS and NSSS groups; (C) comparison of SSD-12 scores between the SSS and NSSS groups; (D) comparison of PHQ-15 scores between the SSS and NSSS groups; and (E) RU-CC, depression, anxiety, and SSD co-morbidities in patients with SSS.

Patients in the SSS group exhibited significantly more severe psychological symptoms compared to the NSSS group with higher scores on the GAD-7 (*p* < 0.001), PHQ-9 (*p* = 0.002), and SSD-12 (*p* < 0.001). SSD was more prevalent and more severe in the SSS group (χ^2^ = 28.682, *p* < 0.001), as reflected by higher PHQ-15 scores (*p* < 0.001; Table 2 and Figure 3A-D).

RU-CC patients in the SSS group exhibited significantly lower C2 and C5 thresholds compared to RU-CC patients in the NSSS group (C2: *p* = 0.004; C5: *p* = 0.004), indicating heightened capsaicin cough sensitivity. Additionally, the SSS group reported lower LCQ total (*p* < 0.001) and all domain scores (Physical: *p* < 0.001; Psychological: *p* < 0.001; Social: *p* = 0.003). However, there was no significant difference in the VAS and cough symptom scores. RU-CC patients in the SSS group exhibited significantly more severe psychological symptoms compared to the NSSS group with higher scores on the GAD-7 (*p* = 0.004), PHQ-9 (*p* = 0.004), SSD-12 (*p* < 0.001), and PHQ-15 scores (*p* < 0.001; Table 3)

**Table 3.** Comparison of characteristics between the NSSS and SSS patients with RU-CC.

	Variables	NSSS (*N* = 25)	SSS (*N* = 43)	Test results
General clinical characteristics	Age (y)	50.64 ± 13.40	48.35 ± 15.53	*t* = 0.616, *p* = 0.540
	Sex (F/M)	17/8	29/14	χ^2^ = 0.002, *p* = 0.962
	BMI (kg/m^2^)	24.50 ± 4.37	24.01 ± 4.32	*t* = 0.448, *p* = 0.655
	Duration (m)	36.00 (32.00)	48.00 (48.00)	Z = −1.631, *p* = 0.103
	Lung function			
	FEV1 predicted (%)	103.67 ± 18.99	92.58 ± 22.11	*t* = 1.429, *p* = 0.165
	FVC predicted (%)	102.43 ± 15.51	97.74 ± 22.07	*t* = 0.657, *p* = 0.517
	FEV1/FVC%	81.23 ± 7.98	80.97 ± 10.42	*t* = 0.076, *p* = 0.940
	FeNO (ppb)	16.40 ± 9.43	20.02 ± 24.58	*t* = −0.706, *p* = 0.483
	IgE (IU/mL)	27.84 ± 26.62	39.82 ± 43.27	*t* = −1.251, *p* = 0.215
	PBEC (×10^9^/L)	0.98 ± 1.26	0.89 ± 1.50	*t* = 0.239, *p* = 0.812
	sEOS%	0 (2)	1 (2)	Z = −1.129, *p* = 0.259
Cough-related assessment	VAS	54.60 ± 24.20	65.23 ± 14.32	*t* = −2.003, *p* = 0.053
	Cough symptom score			
	Daytime	3.00 (1.00)	3.00 (1.00)	Z = −0.126, *p* = 0.900
	Nighttime	2.00 (2.00)	2.00 (1.00)	Z = −0.410, *p* = 0.681
	Capsaicin cough sensitivity			
	lgC2	1.18 ± 0.52	0.84 ± 0.19*	*t* = 3.136, *p* = 0.004
	lgC5	1.37 ± 0.62	0.96 ± 0.33*	*t* = 3.115, *p* = 0.004
	LCQ score			
	Total score	14.21 (6.17)	10.71 (4.48)**	Z = −3.606, *p* < 0.001
	Physical domain	5.00 (2.12)	3.88 (1.38)**	Z = −3.539, *p* < 0.001
	Psychological domain	4.86 (2.42)	3.57 (1.57)**	Z = −3.493, *p* < 0.001
	Social domain	4.86 (2.43)	3.57 (1.86)*	Z = −3.022, *p* = 0.003
Psychological assessment	SSD-12 score	3.0 (13.5)	16.0 (12.0)**	Z = −4.029, *p* < 0.001
	PHQ-15 score	1.0 (3.5)	6.0 (5.0)**	Z = −4.373, *p* < 0.001
	GAD-7 score	2.0 (4.0)	5.0 (5.0)*	Z = −2.855, *p* = 0.004
	PHQ-9 score	2.0 (4.0)	4.0 (5.0)*	Z = −2.879, *p* = 0.004

Data are presented as the mean ± SD, median (interquartile range), or No. (%) unless otherwise indicated.

RU-CC, refractory chronic cough or unexplained chronic cough; NSSS, non-sensitive skin syndrome; SSS, sensitive skin syndrome; VAS, cough severity visual analog scale; C2, capsaicin solution concentration with ≥ 2 coughs; C5, capsaicin solution concentration with ≥ 5 coughs; SSD − 12, somatic symptom disorder‐B criteria scale; LCQ, Leicester cough questionnaire; PHQ-15, Patient Health Questionnaire‐15; PHQ-9, Patient Health Questionnaire-9; GAD-7, General Anxiety Disorder-7.

*Compared to the NSSS group, *p* < 0.05; **compared to the NSSS group, *p* < 0.001.

A Venn diagram was used to illustrate the overlap among SSS, RU-CC, SSD, anxiety, and depression. The results demonstrated that a substantial number of patients exhibited multiple overlapping conditions. Among patients with SSS, 71 (79.78%) were shown to have at least one psychological disorder, including 45 (50.56%) with SSD, 37 (42.70%) with anxiety, and 37 (41.57%) with depression. The largest intersection occurred between SSS, RU-CC, and SSD (30 patients), suggesting a close association between SSS, RU-CC, and somatic symptom burden (Figure 3E).

### Treatment response to neuromodulators in RU-CC patients with and without SSS

**Figure 4.**
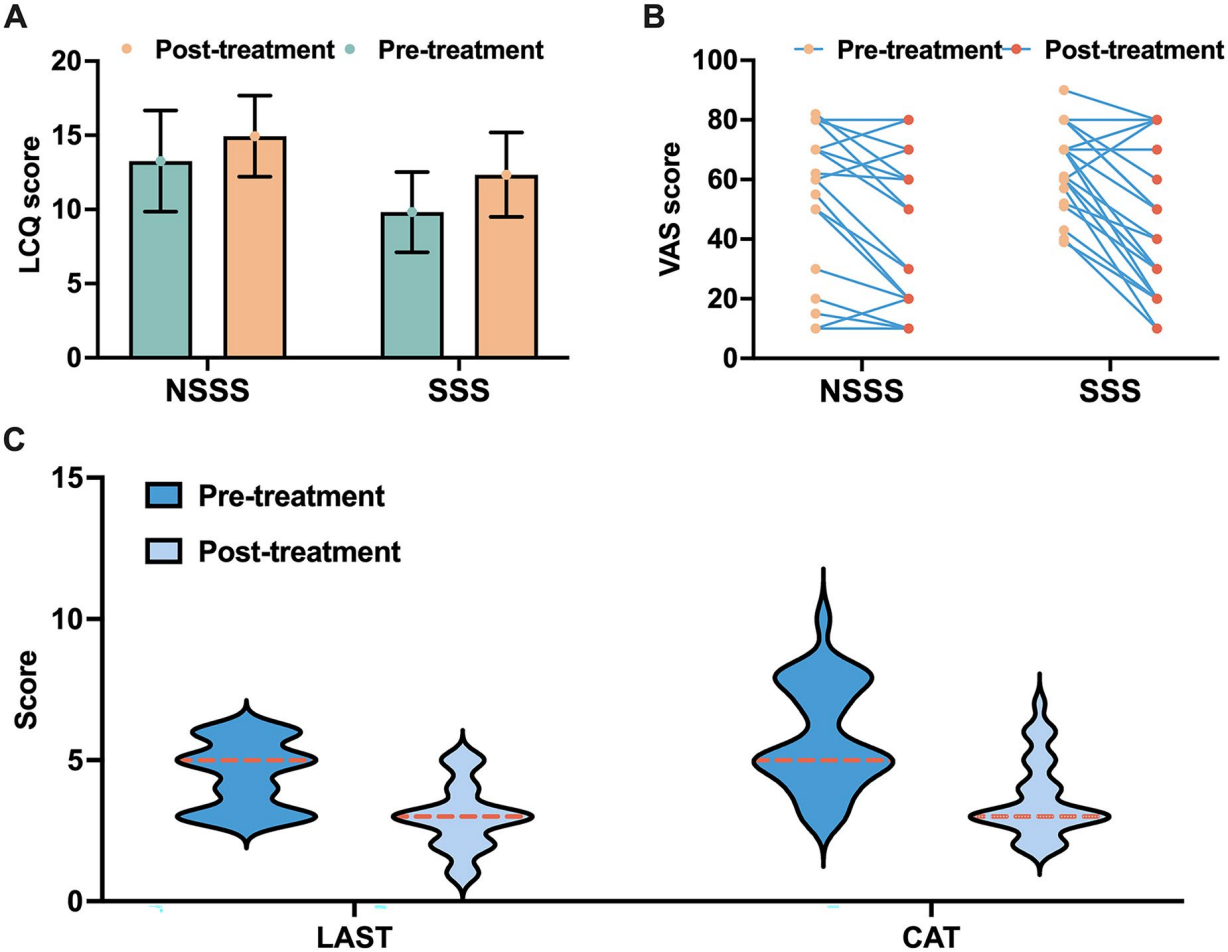
Changes in CHS and SSS evaluations before and after neuromodulator treatment in patients with RU-CC. (A) Changes in LCQ scores before and after treatment in patients with RU-CC in the SSS and NSSS groups; (B) changes in VAS scores before and after treatment in patients with RU-CC in the SSS and NSSS groups; and (C) changes in LAST and CAT scores before and after treatment in RU-CC patients with SSS.

RU-CC patients were followed without altering the existing treatment regimens. Six patients declined evaluations and were lost to follow-up. Treatment efficacy was determined using the minimal clinically important difference (MCID) of the LCQ, with a change in LCQ score (ΔLCQ) ≥ 1.3 considered clinically effective [35]. Among the remaining patients, 15 (10 in the SSS group and 5 in the NSSS group) exhibited symptom improvement following a 2-week course of double-dose proton pump inhibitor therapy. The remaining 47 patients (29 in the SSS group and 18 in the NSSS group) received neuromodulator therapy and had follow-up evaluations after 4 weeks of treatment. The mean change in the ΔLCQ in the SSS group was significantly greater than the NSSS group (2.59 ± 2.36 vs. 1.26 ± 2.53; *t* = −1.832, *p* = 0.037). The overall response rate was also significantly higher in the SSS group compared to the NSSS group (79.3% vs. 44.4%; χ^2^ = 4.748, *p* = 0.029; Figure 4A).

The VAS score showed a significant reduction after treatment compared to baseline in the SSS group (65.24 ± 13.92 vs. 42.41 ± 22.78; *t* = 4.604, *p* < 0.001), whereas the change in the VAS score was not statistically significant in the NSSS group (53.00 ± 25.27 vs. 41.67 ± 25.26; *t* = 1.346, *p* = 0.094; Figure 4B).

**Figure 5.**
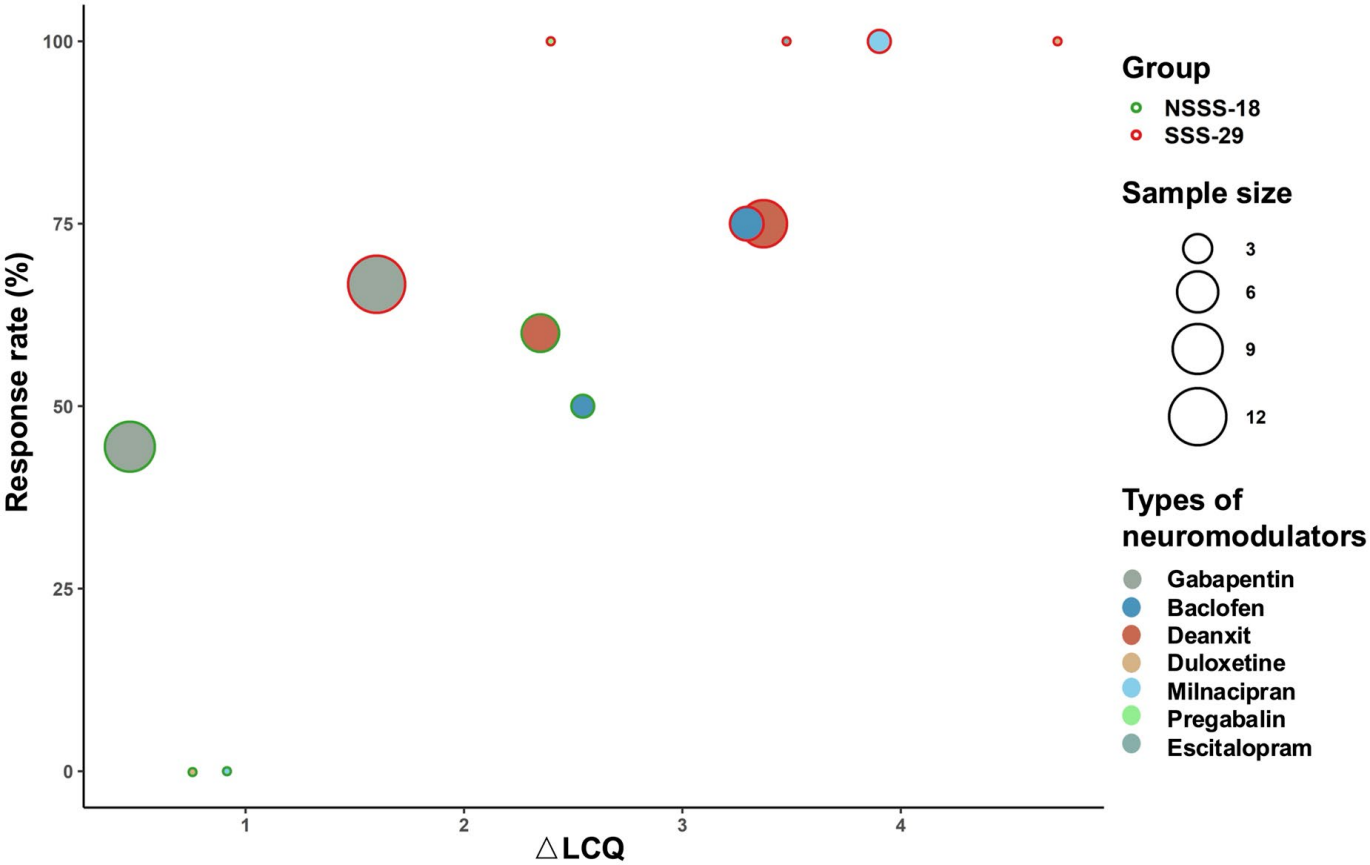
Bubble chart of neuromodulator therapy in the two groups. Bubble colours represent different medications and bubble radius indicates sample size.

After follow-up treatment with neuromodulators in 29 patients with RU-CC combined with SSS, the SSS scores significantly decreased compared to pre-treatment (LAST: Z = −4.177, *p* < 0.001; CAT: Z = −4.328, *p* < 0.001), indicating an improvement in SSS symptoms after treatment (Figure 4C). Details regarding neuromodulator use and changes in VAS and LCQ scores before and after treatment are presented in Figure 5.

## Discussion

This prospective study is the first to systematically evaluate the comorbidity of SSS in CC patients using dermatologic, respiratory, and psychosomatic assessments. Our results demonstrated that SSS was identified in 44.5% of CC patients in our cohort, and this proportion was higher (63.24%) in the subgroup of patients with refractory or unexplained chronic cough (RU-CC). Furthermore, CC patients with co-morbid SSS exhibited a distinct clinical profile marked by heightened cough reflex sensitivity, greater perceived cough severity, significantly impaired quality of life, and a heavier psychological burden. In this exploratory analysis, the presence of SSS was associated with a greater improvement in quality of life and a higher response rate following neuromodulator therapy in RU-CC patients. These results collectively suggested that SSS identifies a clinically relevant phenotype within the CC population, potentially underpinned by shared mechanisms of neural hypersensitivity.

The observed co-morbidity between SSS and CC, particularly RU-CC, strongly suggested an overlap in the underlying pathophysiology. While a prior population-based study reported a similar association, our findings, derived from a clinical cohort using standardised diagnostics, provide more robust evidence and minimise the bias inherent in subjective self-reports [18]. The specific nature of the skin symptoms in our cohort is highly instructive. Itching (59.55%) and pain (39.33%) were the most prevalent SSS symptoms, mirroring findings from a large European survey that linked cough to these specific unpleasant skin sensations [21]. The co-occurrence of these symptoms points beyond simple association towards possible shared neurobiological pathways. The higher burden of allergic rhinitis and atopic dermatitis/urticaria in our SSS patients provides a clinically relevant context for this shared neurobiology. Both conditions are characterised by type 2 inflammation and the release of neuroactive mediators such as histamine, interleukins, and proteases that can directly sensitise sensory nerve fibres such as TRPV1+, TRPA1+ neurons. This persistent peripheral sensitisation in the nasal and cutaneous compartments could prime or amplify similar pathological processes in the airway cough pathways, facilitating a state of generalised sensory hyperresponsiveness across multiple epithelial surfaces. Indeed, a growing body of evidence posits that CC, pruritus, and pain, all being protective senses that can become pathologic, engage analogous peripheral and central neural circuits [16,35].

At the peripheral level, a common pathophysiologic basis lies in the sensitization of sensory nerves. The perception of cough, itch, and pain is mediated by myelinated Aδ and unmyelinated C-fibres [1,36,37]. In chronic conditions, these nociceptive pathways can undergo sensitisation, leading to heightened responsiveness. Crucially, key molecular receptors implicated in cutaneous itch and pain are also integral to the cough reflex. For example, TRPA1 and TRPV4 channels, known for roles in neurogenic itch and pruritic skin diseases [38–40], are also established mediators of cough hypersensitivity [41,42]. Similarly, the Nav1.7 sodium channel and P2X3 receptor, vital for itch and pain transduction [43–46], are emerging targets in cough research. The involvement of opioid receptors, which modulate cutaneous neuro-immune responses [47,48], in chronic cough therapy further underscores this mechanistic overlap [49]. Peripheral sensitisation may thus represent a common pathophysiologic basis for chronic itch, pain, and cough, potentially facilitating cross-sensitisation across different disease states.

Centrally, these sensory signals are integrated and modulated to generate perception and behavioral responses. Functional MRI studies have revealed significant overlap in brain regions activated by pain and cough-evoking stimuli, including the insular cortex, anterior cingulate cortex (ACC), primary sensory cortex, midbrain, and cerebellum [50,51]. Recent animal studies further support the interconnected central mechanisms [52]. Similarly, chronic itch is associated with altered brain activity in regions such as the ACC, posterior cingulate cortex, and prefrontal areas, along with structural and functional reorganization within cortical networks, manifesting as reduced grey matter volume/density and disrupted functional connectivity [53]. This finding suggests that itch, pain, and cough may share common central neural networks or pathways, providing a structural basis for the interaction. Although cough symptoms did not differ significantly between the SSS and NSSS groups, cough-related quality of life was lower in SSS patients, as evidenced by lower scores in psychological domains and highlighting the importance of psychological factors in this subgroup. Multiple studies corroborate the strong association between SSS and psychological co-morbidities, like anxiety and depression [54]. This finding may stem from the negative life impact of skin symptoms and heightened neural sensitivity in these patients. Although anxiety and depression are known co-morbidities in CC, greater than one-half of the CC patients with SSS met the criteria for SSD, indicating not merely emotional distress from symptoms but also catastrophizing cognitions amplifying discomfort. This finding further implicated neural sensitization.

Interestingly, our analysis revealed distinct etiological patterns and temporal relationships in patients with cough-SSS comorbidity, suggesting heterogeneous underlying mechanisms. In non-RU-CC patients, SSS was predominantly associated with conditions linked to inflammation and atopy such as CVA, AC and UACS, where skin symptoms frequently preceded or coincided with cough onset, a pattern consistent with a shared systemic inflammatory diathesis. While, among RU-CC patients, SSS was primarily observed in refractory GERC and UCC, often following a “cough-first” temporal pattern, implying a sequential or concurrent neural sensitisation process. These findings delineate two clinical phenotypes within the cough-SSS spectrum: an “inflammatory-atopic” phenotype and a “neurogenic” phenotype. Therefore, a careful assessment of both cough etiology and the temporal history of skin symptoms in patients with CC can serve as a simple yet insightful clinical tool for phenotyping and guiding personalised treatment strategies.

The “neurogenic” phenotype, characteristic of RU-CC patients with comorbid SSS typically lacks elevated classic inflammatory markers, such as blood eosinophils, IgE, or FeNO, yet exhibits a better response to neuromodulator therapy. The concurrent improvement in both cough and skin symptoms following such treatment underscores a shared, nerve-centric pathophysiologic mechanism. This leads us to hypothesise that these patients may suffer from a generalised neural hypersensitivity syndrome, extending beyond the airways to involve both peripheral and central sensory processing, manifesting as multisensory hypersensitivity. However, future high-quality trials are needed to determine the most effective therapeutic regimens, which may extend beyond first-line gabapentin to include opioids, antidepressants, anxiolytics, or the combination with non-pharmacologic approaches.

Our study has several limitations. The single-center design and relatively small sample size, particularly within the RU-CC subgroup receiving neuromodulators, limit the generalizability of our findings and precluded adjusted analyses for potential confounders. Additionally, as an observational study, patients’ background treatments for cough prior to and during the study were not standardized or adjusted for, which may confound the interpretation of neuromodulator response. The absence of a control group also prevents definitive conclusions about whether SSS prevalence in CC exceeds that of the general population. Furthermore, symptom assessments lacked objective measures, such as confocal skin microscopy to evaluate cutaneous nerve fibers in SSS and 24-h cough monitoring for objective cough counts. Therefore, our preliminary findings require validation in larger, prospective, and well-controlled studies to confirm the association between SSS and cough hypersensitivity phenotypes and to evaluate the efficacy of targeted neuromodulator therapies.

## Conclusion

This study suggests that sensitive skin syndrome is a prevalent and clinically significant co-morbidity in chronic cough, particularly in refractory or unexplained cases. Our findings indicate that the presence of SSS may serve as a clinical biomarker for generalised neural hypersensitivity and is associated with a better response to neuromodulator therapy in refractory patients in this cohort. Early recognition of SSS could thus help guide more targeted treatment strategies.

## Supplementary Material

Supplementary Table.docx

## Data Availability

Some or all datasets generated during and/or analyzed during the current study are not publicly available but are available from the author (TYZZ) on reasonable request.

## Authors approval

All authors have read and approved the final version of the manuscript.

## Open scholarship

This article has earned the Center for Open Science badges for Open Data and Preregistered. The data and materials are openly accessible at 10.5281/zenodo.18654183 and http://www.chictr.org.cn/[ChiCTR2400083115].
