# Supplementary material for: Skin hypersensitivity in chronic cough patients: symptom profiles and psychosomatic correlates
Source: Ann Med. 2026 Mar 9;58(1):2638047. doi: 10.1080/07853890.2026.2638047 (PMC12973777; doi:10.1080/07853890.2026.2638047)
Supplement: Supplementary Table.docx [file IANN_A_2638047_SM6386.docx]

**Supplementary Table 1.** Comorbidities in the study cohort

| **Comorbidity** | **Overall n (%)**  **(N=200)** | **NSSS n (%)**  **(N=111)** | **SSS n (%)**  **(N=89)** | **Test results** |
| --- | --- | --- | --- | --- |
| Allergic rhinitis / Chronic rhinosinusitis | 51 (25.5) | 21 (18.9) | 30 (33.7) ^*^ | χ^2^=5.687, *P*=0.017 |
| Allergic skin diseases^a^ | 29 (14.5) | 0 (0) | 29 (32.6) ^**^ | χ^2^=39.715, *P*<0.001 |
| Hypertension | 63 (31.5) | 31 (27.9) | 32 (36.0) | χ^2^=1.475, *P*=0.225 |
| Diabetes mellitus | 33 (16.5) | 20 (18.0) | 13 (14.6) | χ^2^=0.407, *P*=0.518 |
| Cardiac diseases^b^ | 28 (14.0) | 17 (15.3) | 11 (12.4) | χ^2^=0.358, *P*=0.549 |
| Thyroid disorders^c^ | 17 (14.0) | 11 (15.3) | 6 (12.4) | χ^2^=0.638, *P*=0.425 |

NSSS, non-sensitive skin syndrome; SSS, sensitive skin syndrome.

^a^ Refers to skin conditions caused by allergic reactions, diagnosed by a dermatologist; ^b^ Refers to primary heart diseases (such as coronary artery disease, heart failure, arrhythmias, valvular heart disease, etc.); ^c^ Not including benign thyroid nodules.

^*^ Compared with NSSS group, *P*<0.05; ^**^ compared with NSSS group, *P*<0.001.
